# Impact of liver damage on blood-borne variables and pulmonary hemodynamic responses to hypoxia and hyperoxia in anesthetized rats

**DOI:** 10.1186/s12872-019-01297-z

**Published:** 2020-01-13

**Authors:** Ali Sepehrinezhad, Amirreza Dehghanian, Ali Rafati, Farzaneh Ketabchi

**Affiliations:** 1 Department of Physiology, School of Medicine, Shiraz University of Medical Sciences, Shiraz, Iran; 2 Department of Pathology, School of Medicine, Shiraz University of Medical Sciences, Shiraz, Iran

**Keywords:** Estradiol, Liver disorder, Platelet, RVSP

## Abstract

**Background:**

Liver disorders may be associated with normal pulmonary hemodynamic, hepatopulmonary syndrome (HPS), or portopulmonary hypertension (POPH). In this study, we aimed to investigate the effect of the severity of liver dysfunctions on blood-borne variables, and pulmonary hemodynamic during repeated ventilation with hyperoxic and hypoxic gases.

**Methods:**

Female Sprague Dawley rats were assigned into four groups of Sham (*n* = 7), portal vein ligation (PPVL, *n* = 7), common bile duct ligation (CBDL, *n* = 7), and combination of them (CBDL+ PPVL, *n* = 7). Twenty-eight days later, right ventricular systolic pressure (RVSP) and systemic blood pressure were recorded in anesthetized animals subjected to repeated maneuvers of hyperoxia (O_2_ 50%) and hypoxia (O_2_ 10%). Besides, we assessed blood parameters and liver histology.

**Results:**

Liver histology score, liver enzymes, WBC and plasma malondialdehyde in the CBDL+PPVL group were higher than those in the CBDL group. Also, the plasma platelet level in the CBDL+PPVL group was lower than those in the other groups. On the other hand, the serum estradiol in the CBDL group was higher than that in the CBDL+PPVL group. All the above parameters in the PPVL group were similar to those in the Sham group. During ventilation with hyperoxia gas, RVSP in the CBDL+PPVL group was higher than the ones in the other groups, and in the CBDL group, it was more than those in the PPVL and Sham groups. Hypoxic pulmonary vasoconstriction (HPV) was not detected in both CBDL+PPVL and CBDL groups, whereas, it retained in the PPVL group.

**Conclusion:**

Severe liver damage increases RVSP in the CBDL+PPVL group linked to the high level of ROS, low levels of serum estradiol and platelets or a combination of them. Furthermore, the high RVSP at the noted group could present a reliable animal model for POPH in female rats.

## Background

Hepatopulmonary syndrome (HPS) and portopulmonary hypertension (POPH) are common complications of liver diseases with high morbidity and mortality around the world. Pulmonary artery pressure decreases in HPS as a result of pulmonary vasodilation and vascularization. In addition, the alveolar-arterial O_2_ difference increases in the patients with HPS due to ventilation-perfusion inequality, intrapulmonary shunt and diffusion limitation [1, 2]. On the other hand, pulmonary artery pressure increases in POPH as a consequence of pulmonary vasoconstriction and vascular remodeling [3–5]. Furthermore, the impairment of gas exchange may occur in POPH patients [6, 7].

The mechanisms of POPH and HPS have not been fully understood yet. It has been proposed that some vasoconstrictive and proliferative substances may escape the liver through porto-systemic shunt, and influence directly the pulmonary smooth muscle cells and capillary endothelium. Also, a prolonged increase in cardiac output may harm the pulmonary capillary endothelium. All aforementioned may pave the way for vascular remodeling and induction of POPH [3, 8, 9]. In addition, the inflammatory cytokines and carbon monoxide may involve in the development of HPS [9, 10]. It has been reported that endothelin-1 increases the expression of endothelial NO synthase and NO production through the activation of endothelin B receptors in HPS [11, 12]. By contrast, endothelin-1 has a vasoconstrictive effect through endothelin A and B receptors in the smooth muscle cells of pulmonary vessels [13]. We previously indicated that ET-B receptors on the pulmonary smooth muscle cells play roles in the regulation of pulmonary vascular tone in animal model of liver cirrhosis [14]. Human studies have shown that reactive oxygen species (ROS) increase in POPH, but not in HPS [6, 15], whereas, ROS are reportedly increased in animal models of HPS [7, 16–18]. Furthermore, high level of ROS may decrease the bioavailability of NO and lead to the constriction of pulmonary vessels [19]. Taken together, all these suggested mechanisms cannot be linked directly to POPH because of lacking an accepted animal model for POPH, and some limitations in human studies [20–22].

It has been reported that the prevalence of pulmonary artery hypertension in women is higher than men [23]. Some scientists link this difference to sexual hormones like estradiol, whereas, others are against this conclusion [24–26]. Also, there are inconsistent studies about the effect of the estradiol receptors of ERα and ERβ in pulmonary hypertension [27]. Besides, plasma concentration of estradiol increases in male and female animals with liver dysfunctions. This may be due to the production of estradiol in the stomach or the lack of its metabolism in the injured liver [28, 29]. However, little attention has been paid to the association of pulmonary hemodynamic with serum estradiol in liver dysfunctions.

There are a few controversial investigations concerning the relationship between the severity of liver disease and pulmonary dysfunctions [30–32]. However, due to the complexity in human studies, the classification of liver damage in patients may not be as accurate as the animal studies. HPS can be developed by a common bile duct ligation or drug administration in animals [7, 18]. Nevertheless, a reliable method has not been introduced for the induction of POPH thus far. Although, POPH can be linked to portal hypertension, the increase in portal pressure by partial mechanical obstruction of the portal vein in animals does not lead to pulmonary hypertension [33]. In addition, the increase of pulmonary artery pressure following the injection of sephadex microsphere into the portal vein [22], creation of porto-systemic shunt [21] and intraperitoneal administration of carbon tetrachloride [20] could not entirely create a condition like POPH in human.

Unlike the systemic circulation, alveolar hypoxia, constricts the vessels in the affected region of the lung, thereby moves the blood from the area with low oxygen pressure to the well- ventilated ones. This physiologic phenomenon is called hypoxic pulmonary vasoconstriction (HPV). The limited studies have reported that HPV decreases or disappears in patients with liver disease and animal model of cirrhosis [34, 35]. Nevertheless, the question remains whether the sensitivity of pulmonary vessels to alveolar hypoxia is influenced by the kind or severity of liver disorders.

With the above background, in this study, we established three graded models of liver dysfunctions including mild (PPVL), moderate (CBDL), and for the first time, severe (CBDL+ PPVL), based on liver histology and blood-borne variables. We assessed pulmonary and systemic hemodynamic, and the sensitivity of pulmonary vessels to hypoxia during repeated ventilation with hyperoxic and hypoxic gases. This study was performed in female rats, because the relationship between the estradiol level and the prevalence of pulmonary hypertension with liver dysfunction has not been reported in female animals yet.

## Methods

### Study design

Twenty eight female Sprague-Dawley rats (200–250 g) were purchased from the Laboratory Animal Breeding Center of Shiraz University of Medical Sciences, Shiraz, Iran. The animals were housed in standard cages under controlled laboratory temperature, humidity, and 12:12 h’s light/dark cycles. They had free access to water and standard food, and were fasted 2 h before starting experiments. Animals were divided randomly into 4 groups of Sham, partial portal vein ligation (PPVL), common bile duct ligation (CBDL), and combinations of them (CBDL+PPVL). In order to rule out effects of diurnal variations on our results, all experiments were performed during 11 am-16 pm. *n* = 7 in each group.

### Evaluation of estrus cycle

Only animals in diestrus cycle were entered to the study to exclude the effect of estrus cycle and different concentrations of estradiol on the experiments. This was performed by analysis the cell types of the vaginal lavages according to the previous studies [36]. Briefly, 10 μl of saline was injected three times in the vagina of the restrained animals, and aspirated using a micropipette (Eppendorf, Germany). One drop of the obtaining liquid was placed evenly on a glass slide and drawn in a thin layer (smear). The slides were then dried at room temperature, fixed with 70% alcohol for 5 min and stained using Giemsa dye. Next, estrus cycle was determined based on the type and distribution of cells in the slides using a light microscope (ZEIZZ, Germany). Diestrus cycle was approved when neutrophil numbers were more than the epithelial cells.

### Compositions of gases

Three different gas mixtures were employed in this study, (i) air, (ii) hyperoxic gas: 50% O_2_ balanced with N_2_ (OX), and (iii) hypoxic gas: 10% O_2_ balanced with N_2_ (HOX) [37].

### Study protocol

The experiments were designed in two phases. At the first phase, animals were anesthetized by intraperitoneal injection of 60 mg/kg Ketamine hydrochloride and 10 mg/kg Xylazine [38]. In addition to anesthesia, Ketamine was used to relieve the pain during, and after the surgeries [39]. The skin, fascia, and muscle layers of the upper abdomen were dissected, and liver was exposed. In the CBDL groups, the upper portion of common bile duct was separated from the connective tissues and ligated entirely [40]. In the PPVL group, portal vein was partially ligated [41]. In the CBDL+PPVL group, combinations of CBDL and PPVL were carried out. After surgeries, muscles, fascia and skin were sutured and the conscious animals were then returned back to the animal house and remained in the vivarium during 28 days. All surgical procedures were performed under aseptic conditions.

**Fig. 1:**
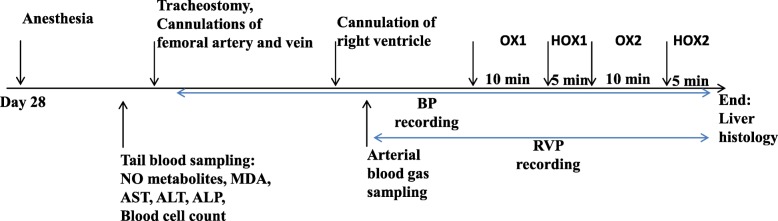
Representative timeline of the study design at day 28 of the experiments. NO: Nitric oxide, MDA: malondialdehyde, AST: aspartate aminotransferase, ALT: alanine aminotransferase, ALP: alkaline phosphatase, OX: ventilation with oxygen 50%, HOX: ventilation with hypoxic gas (oxygen 10%), BP: systemic blood pressure and RVP: right ventricular pressure

The second phase of experiments was performed 28 days later. Animals were anesthetized by intraperitoneal injection of 60 mg/kg sodium thiopental [38, 42]. It is important to emphasize that barbiturates have protective effects on the cardiovascular system, and has been preferred compared to other anesthetics like ketamine during hemodynamic measurements [43, 44]. One point five ml of tail blood sample was taken in micro tubes containing ethylenediaminetetraacetic acid (Sigma) and centrifuged at 1200 rpm for 3 min. Plasma was separated and stored at − 70 °C in order to measure aspartate aminotransferase (AST), alanine aminotransferase (ALT) and alkaline phosphatase (ALP), malondialdehyde (MDA) and NO metabolites (nitrie+nitrate). Also, 0.5 ml of the blood was taken for white blood cell count (WBC) and serum estradiol measurement. A tracheostomy and mechanical ventilation was achieved (Palmer, England). Then, the femoral artery and vein were cannulated. Right ventricular catheterization was executed according to the procedures of the previous studies [14, 38]. Mean systemic arterial blood pressure (mBP), right ventricular systolic pressure (RVSP), as an indicator of pulmonary systolic pressure; and right ventricular diastolic pressure (RVDP) were recorded through the cannula of the femoral artery and right ventricle connected to a data acquisition system (Powerlab, AD instruments Australia). After a steady state period, arterial blood gases and pH were measured by blood samples taken from the femoral artery cannula. Then, animals were ventilated with hyperoxic gas (50% O_2_) for 10 min, followed by 5 min ventilation with hypoxic gas (10% O_2_). Next, 10 min ventilation with hyperoxic gas and 5 min ventilation with hypoxic gas was repeated. The method of hypoxic challenges was optimized from our previous studies and others [38, 45, 46]. At the end of experiments, anesthetized animals were killed by repeated intravenous injection of Ketamine (60 mg/kg/0.5 ml saline) through the catheter of femoral vein. The liver was removed and prepared for histological examinations. Figure 1 shows a representative timeline of the study design at day 28.

### Measurements

Plasma was analyzed for AST, ALT and ALP spectrophotometrically using an automated analyzer system (DIRUI CS-1200, China). Blood gas variables were measured by Easylyte blood gas analyzer (Medica, USA). Also, WBC and platelet count were measured by autoanalzing method (KX-21, Sysmex, Japan). Serum estradiol was measured by ELISA kit using a micro plate reader (Biotek, USA). All noted measurements were performed in a blinded manner in the hospital.

### Liver histology

At the end of the experiments, the abdomen was opened by a midline incision, liver removed and prepared for histological analyses in the department of pathology. Briefly, livers were fixed using 10% formalin for a few days. Then, the samples were dehydrated by different concentrations of ethanol, and embedded in paraffin. Next, 5 μm of sections were mounted on glass slides and stained with hematoxylin and eosin. All slides of histology were evaluated in a blinded manner by a pathologist that used Ishak histology scoring system for the evaluation of liver histological score in terms of liver necrosis, apoptosis, and inflammation and ductular reaction [47].

### MDA assay

Plasma concentration of MDA was determined using a TBARS assay. Briefly, plasma samples and standards (1,1,3,3-tetraethoxy propane, Sigma) were mixed with 0.25 N, HCl (Sigma), 20% tricholoro acetic acid (Sigma) and 0.8% tribarbituric acid (Sigma) incubated at 90 °C for 60 min, cold in ice, and centrifuged at 4000 rpm for 10 min. Then, 200 μl of prepared solutions was added to each well, and the absorbance of well-plate was read at 532 nm using a microplate reader (Biotek, USA) [48].

### NO metabolites assay

Griess method was used to measure plasma concentration of NO metabolites as described previously [49]. Briefly, plasma samples and standards (sodium nitrite, Sigma) were mixed with N-(1-Naphthyl) ethylenediamine dihydrochloride (NEDD, Sigma), sulfanilamide (SULF, Sigma), and vanadium chloride (Sigma) and incubated at 37 °C for 30 min. Then, 100 μl of prepared solutions was added to each well, and the absorbance of well-plate was detected at 540 nm.

### Statistical analysis

Data are given as mean ± SE. Analysis of variance (ANOVA) with the Tukey’s post hoc test was used for multiple comparisons. We also used the paired T-Test for comparing the data of one group before and after hypoxia maneuvers. Furthermore, we compared data of histology by non-parametric Mann-Whitney test. All analyses were performed using a software of SPSS 18. Significance was assumed when *P* < 0.05 and the confidence limits used were the 95% intervals.

## Results

### Plasma biochemical, blood gas parameters and cell count

AST and ALP in the CBDL and CBDL+PPVL groups were higher than those in the Sham and PPVL groups (*P* < 0.01). Also, ALT in the CBDL+PPVL group was higher than the ones in the Sham and PPVL groups (*P* < 0.01), and in the CBDL group was higher than that in the Sham group (*P* < 0.05). Although, AST, ALP and ALT in the CBDL+PPVL seem to be more than those in the CBDL group, these differences were not statistically significant. In addition, AST, ALP and ALT in the PPVL were similar to those in the Sham group (Table 1).

**Table 1:** Blood parameters in the experimental groups

	Sham	PPVL	CBDL	CBDL+PPVL
ALT (U/L)	54.14 ± 5.1	69.7 ± 10.9	121.8 ± 19.1*	154.1 ± 18.78 **##
AST (U/L)	88.77 ± 5.87	104.57 ± 8.86	486 ± 45.5 **##	610.29 ± 62.55**##
ALP (U/L)	40.85 ± 10.4	31.57 ± 10.26	164.3 ± 11.9**##	203.8 ± 36.05**##
PaO2 (mmHg)	61.4 ± 2.57	63.8 ± 0.75	58.6 ± 3.23	50.8 ± 1.84 *#†
PaCO_2_ (mmHg)	36.38 ± 1.55	32.7 ± 0.51	27.98 ± 2.36 *	40.9 ± 1.47
HCO_3_^−^ (mmol/L)	24.04 ± 0.72	22.9 ± 0.39	22.84 ± 0.99	27.18 ± 0.60*#†
pH	7.46 ± 0.01	7.44 ± 0.01	7.52 ± 0.02 *#	7.43 ± 0.00 †
PaO_2_/FIO_2_	292.4 ± 12.26	303.80 ± 3.59	279.04 ± 15.39	241.90 ± 8.79 *#†
WBC (10^3^/μl)	5.52 ± 0.12	3.96 ± 0.67	13.52 ± 1.55 **##	16.42 ± 0.59 **##†
PLT(103/μl)	532.9 ± 25.5	386.46 ± 28.7	465.7 ± 33.8	244 ± 36.15** #††

*n* = 7 in each group. Data are presented as mean ± SE. **(*P* < 0.01) and * (*P* < 0.05) vs. the Sham group., ## (*P* < 0.01) vs. the PPVL group, †† (*P* < 0.01), and † (*P* < 0.05) vs. the CBDL group. The blood samples were taken at the end of the steady state period while animals were ventilated with room air. The atmospheric pressure is 630 mmHg. *AST* aspartate aminotransferase, *ALT* alanine aminotransferase, *ALP* alkaline phosphatase, *WBC* white blood cell count and *PLT* Platelet

Because the atmospheric pressure is about 630 mmHg at the site where the experiments were conducted, PaO_2_ of 65 ± 5 mmHg and PaO_2_/FIO_2_ ratio of about 300 ± 10 mmHg are considered as normal. PaO_2_ in the CBDL+PPVL group was lower than the ones in the CBDL, PPVL and Sham groups (*P* < 0.05). There was no difference in PaO_2_ values among the CBDL, PPVL, and Sham groups. PaO_2_/FIO_2_ ratio in the CBDL+PPVL group was lower than the ones in the CBDL, PPVL and Sham groups (*P* < 0.05). Although, PaO_2_/FIO_2_ in the CBDL group seems to be lower than those in the PPVL and Sham groups, these differences were not significant. There was no alteration in PaCO_2_ values among the CBDL+PPVL, PPVL, and Sham groups. Nevertheless, PaCO_2_ in the CBDL group was lower than that in the Sham group (*P* < 0.05). Also, pH in the CBDL group was higher than those in the Sham and PPVL group (*P* < 0.05). pH in other group were almost similar. HCO_3_^−^ in the CBDL+PPVL group was higher than the ones in the other groups (*P* < 0.05) (Table 1). Also, there were no significant variation in HCO_3_^−^ among the CBDL, PPVL, and Sham groups.

WBC in the CBDL+PPVL group was higher than those in the CBDL (*P* < 0.05), PPVL and Sham (*P* < 0.01) groups. Also, in the CBDL group, it was higher than the ones in the Sham and PPVL groups (*p* < 0.01). However, there was no difference in WBC between the PPVL and Sham groups. The plasma platelet level in the CBDL+PPVL group was lower than those in the other groups (*P* < 0.01), whereas, there was no difference in platelet level among the CBDL, PPVL and Sham groups (Table 1).

### Liver histology score

**Fig. 2:**
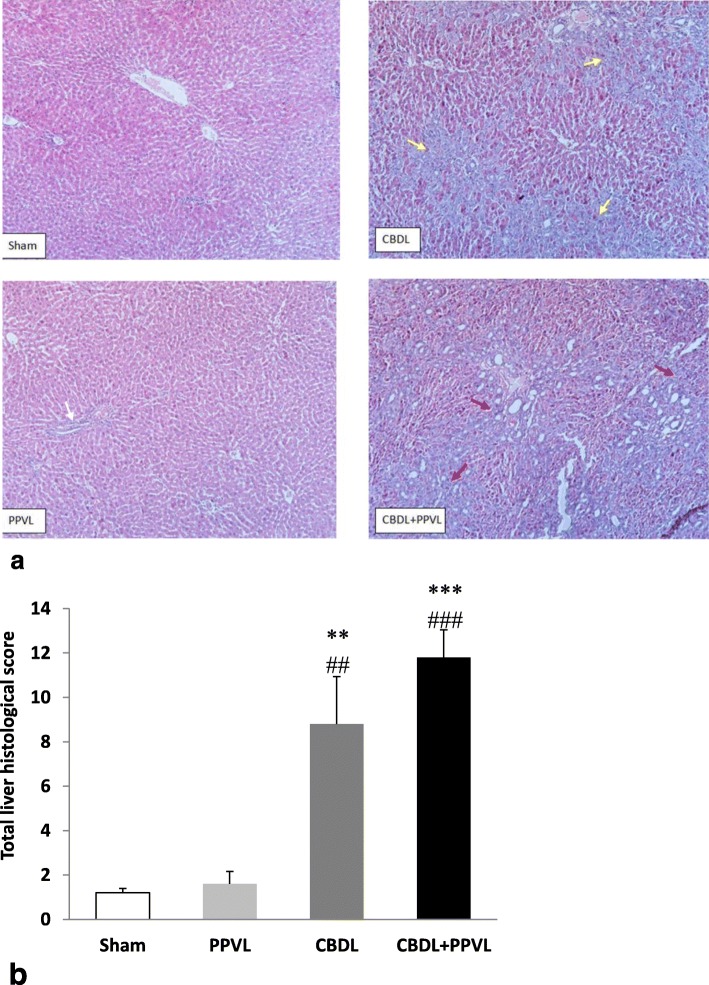
Representative photomicrographs of liver sections stained with hematoxylin & eosin (H&E) in the experimental groups with magnification of 10X. Yellow arrows indicate ductular reaction, white arrows indicate inflammation of the portal vein and red arrows are indicative of fibrosis (**a**). Comparison of *n* = 7 data in each group (**b**). Data are presented as mean ± SE. ***(*P* < 0.001), ** (*P* < 0.01) vs. the Sham group., ### (*P* < 0.001) and ## (*P* < 0.01) vs. the PPVL group

The liver histological score in the CBDL+PPVL group was higher than those in the PPVL and Sham groups (*P* < 0.001). Also, in the CBDL group, it was higher than those in the PPVL and Sham groups (*P* < 0.01). There was no difference in the liver histological scores between the CBDL and CBDL+PPVL groups, or between the PPVL and Sham groups (Fig. 2a, b).

### Hemodynamic measurements

RVSP during the first ventilation with hyperoxia gas (OX1) in the CBDL (*p* < 0.01) and CBDL+PPVL (*p* < 0.001) groups were higher than the one in the Sham group. Also, RVSP in the CBDL+PPVL group was higher than that in the PPVL group (*p* < 0.01). However, there was no significant variation in RVSP between the CBDL and PPVL groups, or between the PPVL and Sham groups.

The first ventilation with hypoxic gas (HOX1) increased RVSP in the Sham and PPVL groups insignificantly with no change in the CBDL and CBDL+PPVL groups. There was no difference in RVSP between the PPVL and Sham groups, or between the CBDL+PPVL and CBDL groups during ventilation with hypoxic gas. Also, there was no difference in RVSP among all groups of Sham, PPVL, CBDL and CBDL+PPVL during ventilation with hypoxic gas.

During ventilation with hyperoxic gas for the second time (OX2), RVSP in the CBDL (*p* < 0.05) and CBDL+PPVL (*p* < 0.01) groups were still higher than that in the Sham group. Also, RVSP in the CBDL+PPVL group was more than that in the PPVL group (*p* < 0.05). However, no alteration in RVSP was detected between the PPVL and Sham groups.

**Fig. 3:**
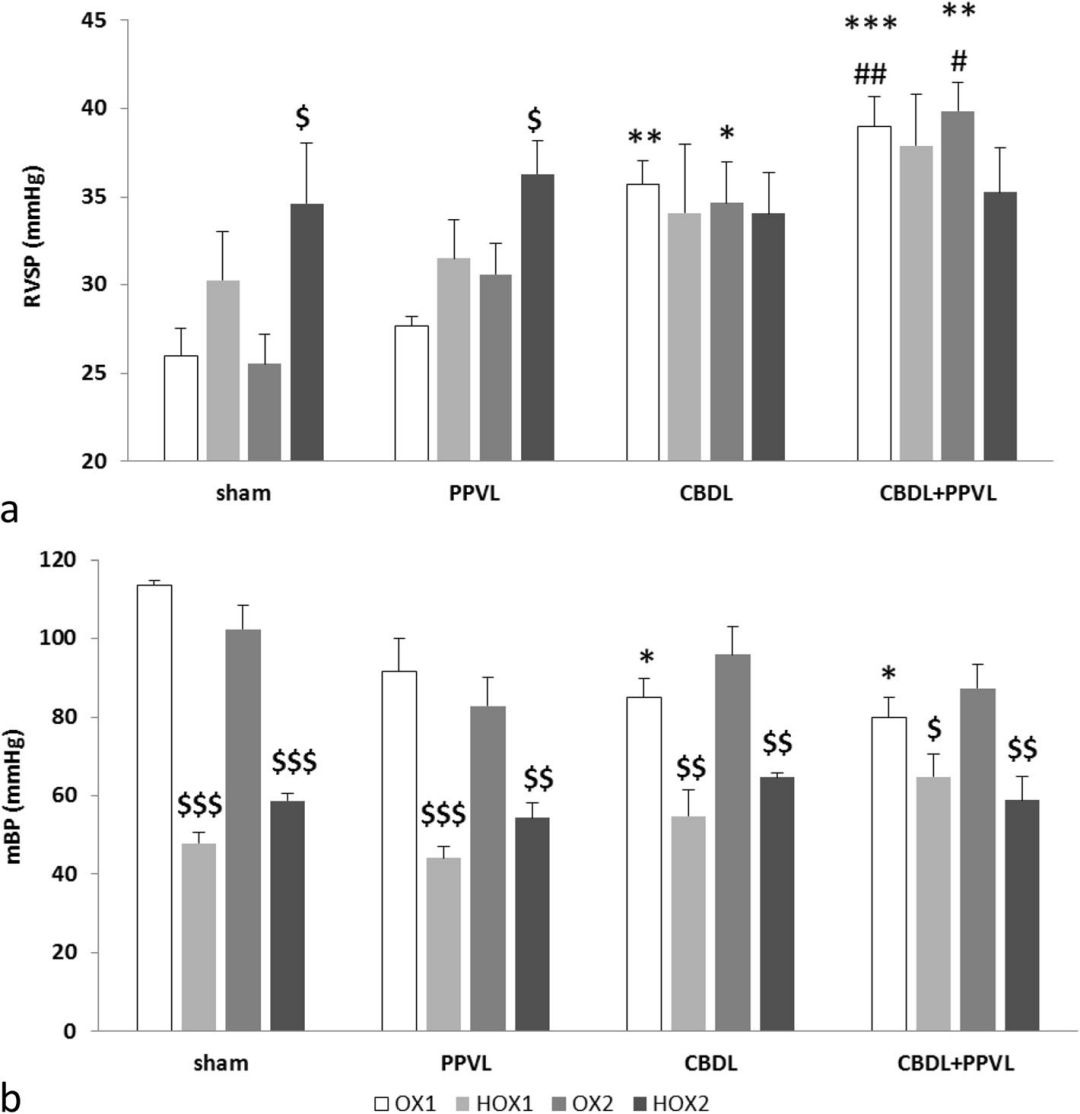
Right ventricular systolic pressures (RVSP) (a) and mean systemic blood pressures (mBP) (b) in the experimental groups before (OX) and after (HOX) hypoxia maneuvers. *n* = 7 in each group. Data are presented as mean ± SE. * (*p* < 0.05); ** (*p* < 0.01); *** (*p* < 0.001) vs. the Sham group., # (*p* < 0.05); ## (*p* < 0.01) vs. the PPVL group., $$$ (*p* < 0.001); $$ (*p* < 0.01) and $ (*p* < 0.05) between OX and HOX conditions

The second hypoxia maneuver (HOX2) increased RVSP in both groups of Sham and PPVL significantly (*p* < 0.05), whereas, RVSP tended to decrease in the CBDL+PPVL group insignificantly. Furthermore, there was no difference in RVSP among all groups of Sham, PPVL, CBDL and CBDL+PPVL (Fig. 3a).

mBP in the CBDL and CBDL+PPVL groups were lower than that in the Sham group (*p* < 0.05) during OX1 conditions. There was no difference in mBP between the PPVL and Sham groups, or between the CBDL and CBDL+PPVL groups. Ventilation of animals with the first and second hypoxia maneuvers decreased mBP in all groups. No alteration was detected between the values of mBP during OX2 conditions (Fig. 3b).

**Fig. 4:**
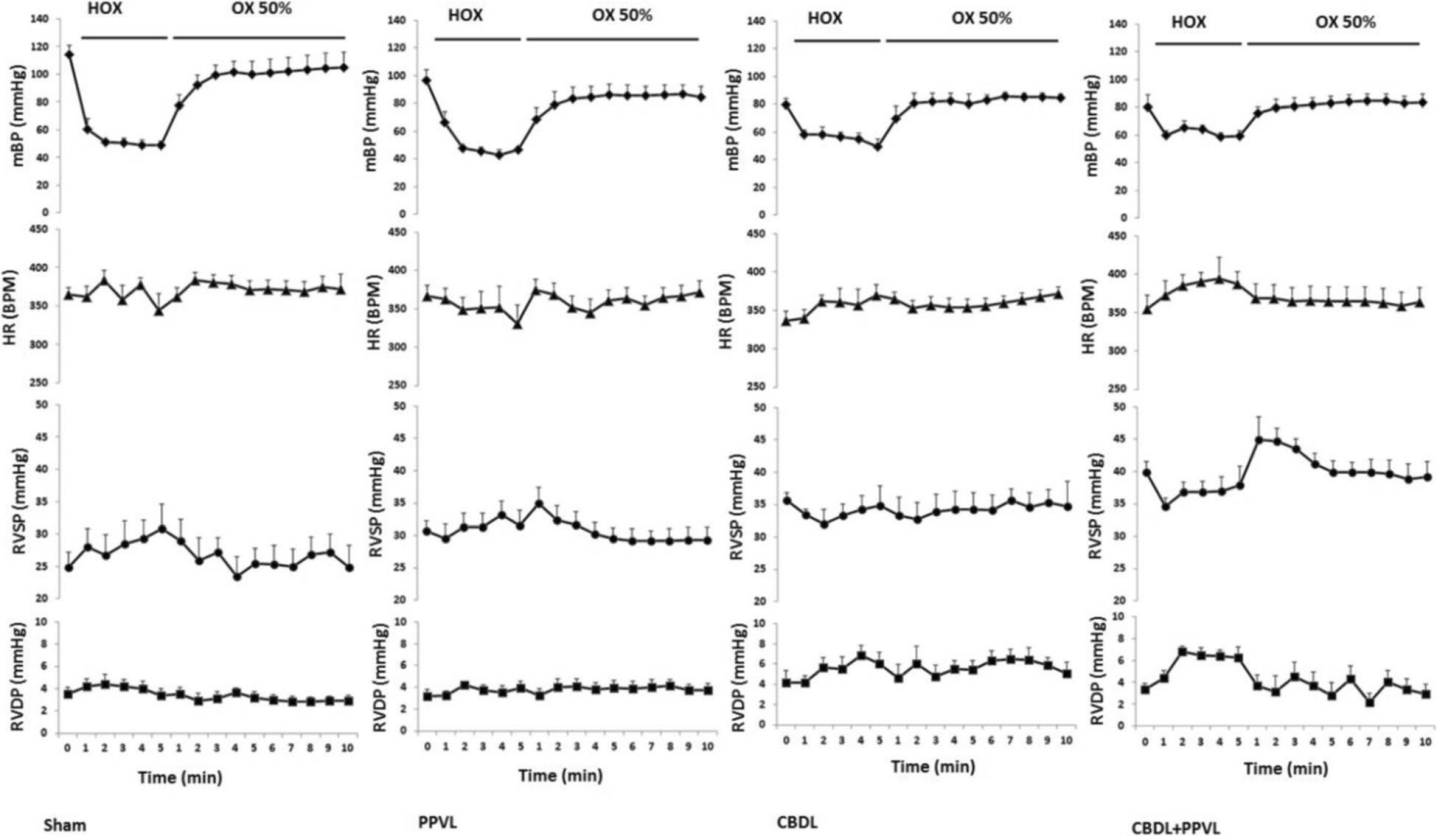
The alterations mean systemic blood pressure (mBP), heart rate (HR), right ventricular systolic pressure (RVSP) and right ventricular diastolic pressure (RVDP) during the time course of experimental groups before hypoxia, during the first hypoxia (HOX) and hyperoxia (OX) maneuvers

The alterations of mBP, heart rate (HR), RVSP and RVDP have been shown in Fig. 4 during the time course of HOX1 and OX2. In the Sham group, mBP decreased substantially being constant during the time course of HOX1 and returned to the values during OX2. There were some oscillations in HR during HOX1 which was augmented during OX2. RVSP increased during HOX1 returning to the values during OX2. RVDP did not alter at before, during or after HOX1. Almost similar results were obtained in the PPVL group. In the CBDL group, HR and RVDP increased, whereas, mBP and RVSP decreased during HOX1. However, the reduction of mBP was not as remarkable as the Sham and PPVL groups. In the CBDL+PPVL group, the alterations of mBP, HR and RVSP were similar to those in the CBDL group during HOX1. However, RVSP increased during OX2. Furthermore, RVDP increased during HOX1, and returned back to its baseline during OX2.

### Plasma MDA

**Fig. 5:**
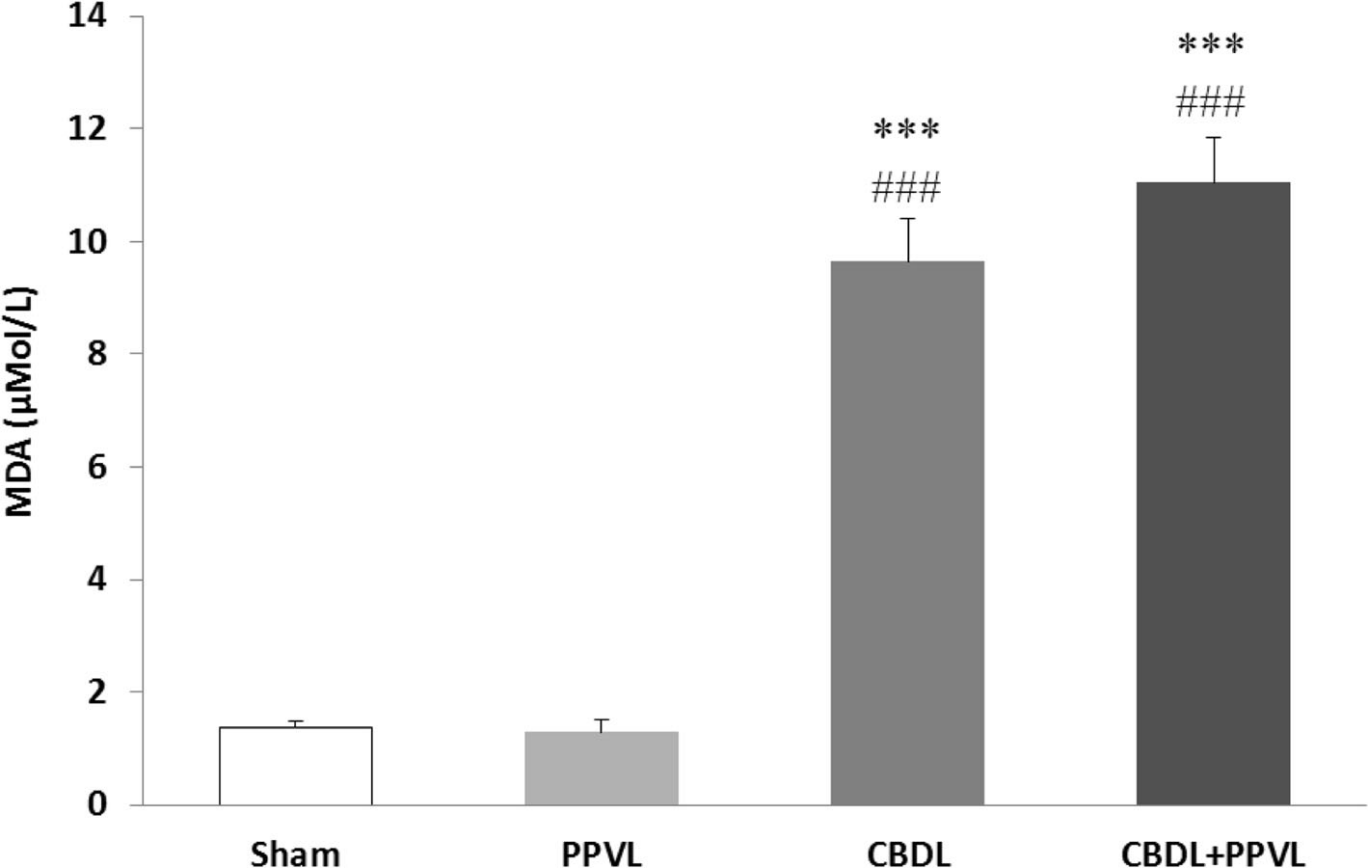
Plasma concentrations of MDA in the experimental groups. *n* = 7 in each group. Data are presented as mean ± SE. *** (*P* < 0.001) vs. the Sham group., ### (*P* < 0.001) vs. the PPVL group

Plasma concentration of MDA in the CBDL and CBDL+PPVL groups were higher than those in the Sham and PPVL groups (*P* < 0.001). There was no significant difference in the values of MDA between the PPVL and sham groups, or between the CBDL and CBDL+PPVL groups (Fig. 5).

### Plasma NO metabolites

**Fig. 6:**
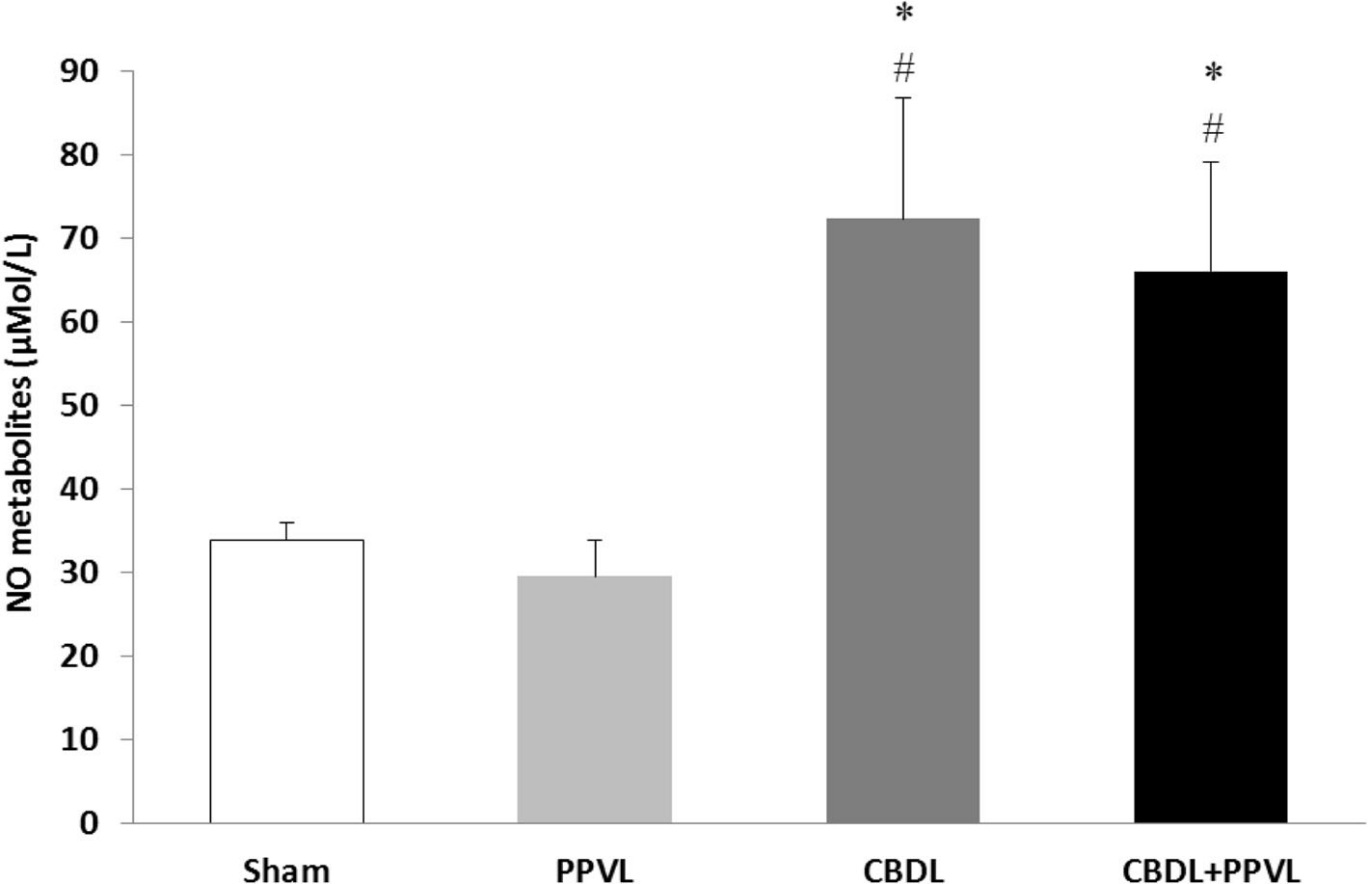
Plasma concentrations of NO metabolites in the experimental groups. *n* = 7 in each group. Data are presented as mean ± SE. * (*P* < 0.05) vs. the Sham group., # (*P* < 0.05) vs. the PPVL group

Plasma concentration of NO metabolites in the CBDL and CBDL+PPVL groups were higher than those in the Sham and PPVL groups (*P* < 0.05). There was no significant difference between the values of NO metabolites between the PPVL and sham groups, or between the CBDL and CBDL+PPVL groups (Fig. 6).

### Serum estradiol

**Fig. 7:**
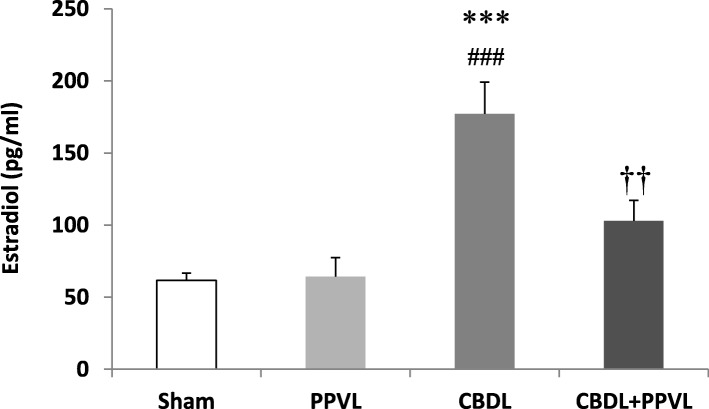
Serum concentrations of estradiol in the experimental groups. *n* = 7 in each group. Data are presented as mean ± SE. *** (*P* < 0.001) vs. the Sham group., ###(*P* < 0.001) vs. the PPVL group., †† (*P* < 0.001) vs. the CBDL group

Serum estradiol concentration in the CBDL group was higher than those in the PPVL and Sham (*p* < 0.001), and CBDL+PPVL (*p* < 0.01) groups. Although estradiol in the CBDL+PPVL group tended to increase, it was not significantly different as compared with the Sham or PPVL groups. Also, estradiol in the CBDL+PPVL group was lower than that in the CBDL group (*p* < 0.01). There was no significant difference in the values of estradiol between the PPVL and sham groups (Fig. 7).

## Discussion

In this study, we developed three graded levels of liver damage based on the liver histology and blood-borne variables and compared RVSP during ventilation with hyperoxic and hypoxic gases. Although the plasma MDA in the CBDL group tended to be lower than that in the CBDL+PPVL group, it was not statistically significant. Furthermore, the enhancement of serum estradiol in the CBDL+PPVL group was less pronounced than the CBDL group. Also, there was a considerable decrease in plasma platelet level in the CBDL+PPVL group. Besides, the impairment of gas exchange through the blood gas barrier occurred only in the CBDL+PPVL group. The results of WBC suggest a substantial inflammatory reaction in the CBDL+PPVL group. Together, we considered the CBDL+PPVL, CBDL, and PPVL groups as models of severe, moderate and mild liver dysfunction, respectively. Though the previous studies have shown the response of pulmonary vessels to hypoxia in liver dysfunction model, we investigated the sensitivity of pulmonary circulation based on the severity of liver damage, and observed the recovery of repeated HPV only in the PPVL group with mild liver dysfunction. We also proposed, for the first time, ligation of both CBDL and PPVL as a possible animal model for induction of POPH.

Although plasma MDA and NO metabolites increased identically in the CBDL and CBDL+ PPVL groups, data of liver histological scores, liver enzymes, low platelet level, and high WBC are indicating a remarkable liver injury and inflammatory reactions in the CBDL+ PPVL group. Therefore, this group was considered as a model for severe liver damage. Furthermore, plasma MDA and NO metabolites, liver enzymes, WBC and liver histology in the CBDL group were higher than those in the PPVL group, and lower than the ones in the CBDL+PPVL group. Consequently, the CBDL group was considered as a model for moderate liver damage. Also, all the above-mentioned variables did not differ between the PPVL and Sham groups which suggests portal vein ligation per se could not lead to a significant liver dysfunction. Therefore, the model of PPVL was considered as a model for mild liver damage. The survival rate of CBDL+PPVL group was almost similar to that in the CBDL group during 28 days of experiments. However, the mortality rate of animals in the CBDL+PPVL groups increases to 50% after 40 days. Therefore, it cannot be recommended for investigating in longer times if untreated.

In order to exclude the effects of different concentrations of estradiol during the estrus cycle, all female animals were entered the study during diestrus phase. High serum estradiol in the CBDL group can be linked to the overproduction of estradiol or the lack of its metabolism in the stomach [28, 29]. On the other hand, estradiol in the CBDL+PPVL group did not increase significantly. This may explain partly the high level of RVSP in this group, as estradiol has preventive effects in pulmonary hypertension induced by monocrtotaline or hypoxia [24, 26, 50, 51]. Therefore, the effects of pulmonary vasoconstrictors may not be counterbalanced substantially by a low estradiol level in the CBDL+PPVL group. As a result, the pulmonary artery hypertension may occur.

High level of RVSPs during ventilation with the first hyperoxic gas in the CBDL+PPVL and CBDL groups were not comparable with other reports. There are a few studies reporting the pulmonary artery pressure or vascular resistance in CBDL model. In one study, pulmonary artery pressure has been measured by inserting a catheter into the pulmonary artery through the umbilical vessel at 2 days before the hemodynamic study in Wistar rats [35]. This may affect the normal conditions of pulmonary hemodynamic. In another study, pulmonary vascular resistance was measured only 2 weeks after induction of CBDL, which may not be enough long for the induction of HPS [22]. However, we measured RVSP directly, by inserting a catheter into the right ventricle, with caution, 28 days after induction of liver dysfunction which can be more accurate compared with other investigations. Since RVSP increased markedly in the CBDL+PPVL group, therefore, it can be suggested that ligations of both portal vein and common bile duct in animals may induce a POPH model in rat. However, we did not measure cardiac output and vascular resistance in this study because of some limitations. Therefore, the increase in RVSP might be caused by volume overload, hyperdynamic circulation, vascular remodeling, or combination of them which must be specified in the further studies.

Little change occurred in RVSP during ventilation of animals with the first and second hypoxic gas in the CBDL+PPVL and CBDL groups. A few data have indicated disruption of pulmonary vascular responses to alveolar hypoxia in cirrhotic patients and conscious animals with cirrhosis [34, 35]. In addition, we evaluated the sensitivity of pulmonary vessels to hypoxia by repeating the hypoxia maneuvers. On the other hand, RVSP increased in the Sham and PPVL groups during ventilation with the first hypoxic gas which were amplified during the second hypoxia maneuver. These data may suggest that elimination of hypoxia response depends on the severity of liver dysfunction. The confirmation is that the hypoxia response in the PPVL group with low liver injury was retained in the second hypoxia maneuver. The increase in RVDP in the CBDl+PPVL group can be related to the damage to the heart and decreased the ability of the heart to tolerate the stress induced by hypoxia. This may also explain the relationship between the severe liver damage with detrimental effects in other organs such as the heart. RVSP decreased in the Sham and PPVL groups during ventilation with hyperoxic gas. However, RVSP increased markedly in the CBDL+PPVL group with no change in the CBDL group during ventilation with hyperoxic gas, which may be related to reducing the bioavailability of NO by oxygen [52].

mBP in the CBDL+PPVL and CBDL groups was lower than that in the Sham group. Since plasma overload and high cardiac output have been reported in liver cirrhosis [53], the low mBP at above mentioned groups can be related to the pronounced effect of peripheral vasodilation relative to the volume overload. The results of mBP in the CBDL groups are consistent with the results of Moezi et al. and Nunes et al. that indicated decreased mBP after 4 and 6 weeks of CBDL in male rats, respectively [35, 54]. Besides, in this study, mBP in the PPVL group was similar to the Sham group due to a mild liver damage. Ventilation of animals with the first and second hypoxic gas decreased mBP roughly in all groups due to the reduction in peripheral vascular resistance [55] which is consistent with the results of Edmunds, et al. that indicated ventilation of animals with 10% oxygen decreases sharply the arterial pressure in male Wistar rats [46]. Also, other studies have indicated the arterial pressure falls during acute hypoxia exposure in animals [56, 57]. Even continuous exposure to hypoxia in conscious animals may decrease both heart rate and systolic blood pressure [58]. However, in our study, the reduced mBP by hypoxic gas was much pronounced as compared with our previous study and some other works in male rats [38, 43]. Sex differences in blood pressure response to hypoxia may explain partly this different response [45]. Also, it is important to mention that in our study, animals were ventilated with hyperoxic gas before each hypoxic maneuver. Ventilation with heperoxic gas constricts the systemic vessels, thereby increases the blood pressure, similar to our study [59]. Therefore, ventilation with hypoxic gas may exacerbate the systemic vasodilatory response leading to a sharp drop in systemic arterial blood pressure. Acute hypoxia in human rises, decreases or doesn’t change the systemic arterial pressure depends on the interaction between sympathetic outflow of chemoreceptors and peripheral vascular resistance [60, 61]. However, the effect of peripheral vasodilation may be principal in our experiments compared with the effect of the sympathetic activity. On the other hand, the intermittent or chronic exposure to hypoxic gas increases blood pressure linked to enhancing the sympathetic activity which is different relative to the acute hypoxic condition in our study [45, 62–64]. It should be noted that alterations of mBP in the CBDL and CBDL+PPVL groups were small during ventilation with hypoxic gas which may be linked to the increase of heart rate and volume overload in cirrhotic animals. Hyperoxic gas recovered the arterial pressure because of direct effect of oxygen on increasing the vascular resistance and reducing the bioavailability of NO. [52, 59]

The heart rate decreased a little (data not shown) at the beginning of hypoxic maneuver linked to a reduction in depolarization rate of cardiac pacemaker cells [65]. It was followed by a tachycardia induced by both sympathetic activity and vagal withdrawal subsequent to the chemo-reflex activity [57]. Also, the heart rate decreased transiently during switching from hypoxia to hyperoxia because of increasing the vagal activity [66].

There was not a difference between the values of PaO_2_ in the CBDL and Sham groups, while PaCO_2_ and pH in the CBDL group were less than those in the Sham group. This could be caused by cirrhosis-induced hyperventilation [67]. On the other hand, low PaO_2_ in the CBDL+ PPVL group may be linked to the dominant effect of diffusion impairment compared with the hyperventilation [67]. Furthermore, a low PaO_2_/FIO_2_ ratio in the CBDL+PPVL group is verifying the injury of the blood gas barrier in the severe liver dysfunction. In both HPS and POPH, low PaO_2_ and low saturation of hemoglobin with oxygen have been reported [6, 7, 68–70]. On the other hand, low PaO_2_ may lead to pulmonary vasoconstriction and explain partly increased RVSP in the CBDL+PPVL group (Table 2).

**Table 2:** The comparison of the experimental variables in the CBDL+PPVL versus CBDL models

CBD + PPVL	Effect	CBDL	Effect
Liver damage ↑↑	Liver dysfunction	Liver damage ↑↑	Liver dysfunction
Liver enzymes ↑↑	Liver dysfunction	Liver enzymes ↑↑	Liver dysfunction
WBC ↑↑	Systemic Inflammation	WBC ↑	Systemic inflammation
PLT ↓↓	Vascular thrombosis	PLT ↔	No change
Basal RVSP ↑↑		Basal RVSP ↑	
HPV	No response	HPV	No response
ROS ↑	Systemic Inflammation	ROS ↑	Systemic Inflammation
PaO_2_ ↓	Impairment of gas exchange	PaO_2_ ↓	Little effect
PaO_2_/FIO_2_ ↓	Impairment of gas exchange	PaO_2_/FIO_2_ ↓	Little effect
Estradiol ↑		Estradiol ↑↑	

The plasma concentration of MDA in the CBDL group was more than those in the sham and PPVL groups. Other investigators also have expressed that oxidants increase and antioxidants decrease in liver cirrhosis [6, 71]. ROS may be involved in HPV [72]. Therefore, the disruption of HPV in the CBDL groups may imply that the pulmonary vasculatures were already maximally stimulated by the observed oxidative stress before hypoxia maneuver. High level of ROS may also lead to vascular remodeling and promotes the pulmonary hypertension [72] which needs to be investigated in the long term (Table 2). High levels of NO metabolites and MDA in both CBDL groups, suggests the productions of large amounts of NO and ROS. The combination of NO and ROS may produce the peroxynitrite, a potent vasoconstrictor oxidant which increases the pulmonary vascular resistance and pulmonary artery pressure [73]. In addition, it can be speculated that a part of the inhibitory response to hypoxia is related to the increase in NO production. There are inconsistent results regarding the NO production in the patients with liver diseases or animal models of cirrhosis. For instance, a few studies have shown that NO production increases in human cirrhosis [74, 75], and NO plays a role in the regulation of pulmonary vascular tone in the animal model of cirrhosis [35]. In contrary, NO synthase inhibitor protein increases, thereby, NO production decreases in the cirrhotic patients [6, 76]. However, we measured the plasma NO metabolites which could release from different sources in the body tissues. Furthermore, high level of NO can be partly linked to high estradiol level in the CBDL groups [77].

The platelet level and RVSP in the CBDL+PPVL group was lower than those in the other groups. It has been indicated that platelet level is linked to pulmonary hypertension and the rate of survival [78, 79]. On the other hand, the level of metaloproteinase decreases in the patients with liver cirrhosis. This enzyme regulates the Von Willberand factor size and platelet adhesive activity. Then, the reduction of the enzymes may lead to platelet deposition in the afferent pulmonary vessels [80]. Furthermore, thrombocytopenia in cirrhosis may be caused by the reduction of hematopoietic growth factor thrombopoietin activity in the liver or platelet sequestration in the spleen [81]. All above possibilities may increase the chance for thrombosis formation and pulmonary hypertension [82]. Therefore, it may be assumed that at least a part of increased RVSP is caused by low platelet level in the CBDL+PPVL group (Table 2).

Together, POPH occurs following liver cirrhosis with portal hypertension, and is associated with a mild hypoxemia, thrombocytopenia and inflammation [1, 6, 81, 83]. In this study, RVSP increased substantially in the CBDL+PPVL group. It was also associated with liver damage and portal hypertension induced by partial portal vein ligation. Furthermore, there was a mild hypoxemia in this group. Also, the platelet level decreased only in this group which could increase the prevalence of pulmonary hypertension following thrombotic abnormality. Therefore, the model of CBDL+PPVL can be suggested as a reliable animal model for POPH.

## Conclusions

In this study, we developed three levels of mild, moderate and severe liver dysfunctions in terms of liver histology and biochemical substances in the blood. We found that the ligations of CBDL together with PPVL in female rats, can be used as an animal model for induction of POPH. Also, the hypoxic response may change RVSP based on the severity of liver dysfunction. However, more studies are needed to clarify whether or not this model is applicable in male rats and other species. Furthermore, further studies are needed to compare the mechanisms of high RVSP in the noted model and POPH in human.

## Data Availability

The datasets used and/or analyzed during the current study are available from the corresponding author on reasonable request.

## Abbreviations

ALP: Alkaline phosphatase
ALT: Alanine aminotransferase
ANOVA: Analysis of variance
AST: Aspartate aminotransferase
CBDL: Common bile duct ligation
FIO_2_: Fractional concentration of oxygen in the inspired gas
HPS: Hepatopulmonary syndrome
HPV: Hypoxic pulmonary vasoconstriction
HR: Heart rate
mBP: Mean systemic blood pressure
MDA: Malondialdehyde
NO: Nitric oxide
PaCO_2_: Arterial carbon dioxide pressure
PaO_2_: Arterial oxygen pressure
PLT: Platelet
POPH: Portopulmonary hypertension
PPVL: Portal vein ligation
ROS: Reactive oxygen species
RVSP: Right ventricular pressure
WBC: White blood cell count
